# Recombination and gene loss occur simultaneously during bacterial horizontal gene transfer

**DOI:** 10.1371/journal.pone.0227987

**Published:** 2020-01-28

**Authors:** Bert Ely

**Affiliations:** Department of Biological Sciences, University of South Carolina, Columbia, South Carolina, United States of America; University of California San Diego, UNITED STATES

## Abstract

Bacteria can acquire new genes by incorporating environmental DNA into their genomes, yet genome sizes stay relatively constant. In nature, gene acquisition is a rare event so it is difficult to observe. However, the *Caulobacter crescentus* CB2A genome contains 114 insertions of genetic material from the closely-related NA1000 strain, providing a unique opportunity to analyze the horizontal transfer of genetic material. Analyses of these insertions led to a new model that involves preferential recombination at non-homologous regions that are flanked by regions of homology and does not involve any mutational processes. The net result is the replacement of segments of the recipient genome instead of the simple addition of genetic material during horizontal gene transfer. Analyses of the genomes of closely related strains of other bacterial and archaea genera, suggested that horizontal gene transfer occurs preferentially in non-homologous regions in these organisms as well. Thus, it appears to be a general phenomenon that prokaryotic horizontal gene transfer occurs preferentially at sites where the incoming DNA contains a non-homologous region that is flanked by regions of homology. Therefore, gene replacement is a common phenomenon during horizontal gene transfer.

## Introduction

Comparisons of closely related bacterial genomes can be used to identify regions where one of the genomes has acquired new genes [1]. However, bacterial genome sizes appear to be relatively constant. Therefore, recent studies have suggested that there must be some mechanism for gene loss to counterbalance these gene acquisitions [2, 3]. Furthermore, several studies have shown that most of these imported genomic regions usually come from closely-related genomes but the precise origin cannot be identified [1, 4–6]. Therefore, it has been difficult to perform detailed analyses of the recombination processes that result in the acquisition of new genes via naturally occurring horizontal gene transfer (HGT).

Recently, I determined the genome sequence of CB2A, a version of *Caulobacter crescentus* strain CB2 that was isolated as a variant that does not produce the 130 Kd S-layer protein [7]. The purpose of the sequencing was to confirm that the CB2A genome had acquired a mutation in the *rsaA* gene that codes for the S-layer protein. However, when I examined the CB2A genome sequence, I was surprised to find that more than 100 segments of the closely-related *C*. *crescentus* NA1000 genome had recombined into the CB2A genome. Thus, the CB2A isolate actually had a hybrid genome. Since I knew the nucleotide sequences of the two parental genomes, I had the unique opportunity to examine more than 100 instances of horizontal gene transfer (HGT) where I knew the identity of the donor strain.

Relative to the NA1000 genome, the CB2 genome contains 73,859 SNPs and 644 one bp insertions or deletions, representing approximately 2% of the shared genome [8]. Since these mutational differences are distributed throughout the two genomes, segments of the two genomes are readily distinguished from each other. Analyses of these recombination events led to a new model for horizontal gene transfer that involves preferential recombination at non-homologous regions that are flanked by regions of homology.

## Methods

### Sequencing the CB2A genome

The sequence of the CB2A genome was determined using Pacific Biosciences (PacBio) sequencing technology and assembled de novo as described previously [8]. Other genome sequences were downloaded from GenBank. Accession numbers: NA1000, CP001340; CB2, CP023313; CB2A, CP034122; CB13, CP023315.

### Genome comparisons

For the initial HGT analysis, the NA1000, CB2, and CB2A genomes were compared in pairwise alignments using progressiveMauve with standard settings [9]. A pairwise comparison of the CB2 and NA1000 revealed the presence of extra DNA segments (minimum 50 bp) that were present in one genome but not in the other. These regions were considered indels since they could have arisen by a simple insertion or deletion event. However, in the majority of cases, reciprocal insertions were observed where each genome had at least 50 bp of DNA that was not present in the other genome. The 50 bp minimum may have resulted in counting some reciprocal insertions as simple indels, but it is difficult to be sure that smaller segments are truly insertions of new DNA. The eight insertions that occurred at the site of a tRNA gene were excluded from the analysis since they are posited to occur by a different mechanism. To perform the analysis, an Excel gap file was exported from the Mauve comparison and gaps less than 50 bp were deleted. The remaining gaps were visually compared to the Mauve alignment and those that were aligned were considered to be reciprocal insertions. For the comparisons of other prokaryotic genera, three related genomes were aligned simultaneously with progressiveMauve using standard settings [9] and non-homologous regions were identified by visual inspection.

## Results and discussion

**Fig 1:**
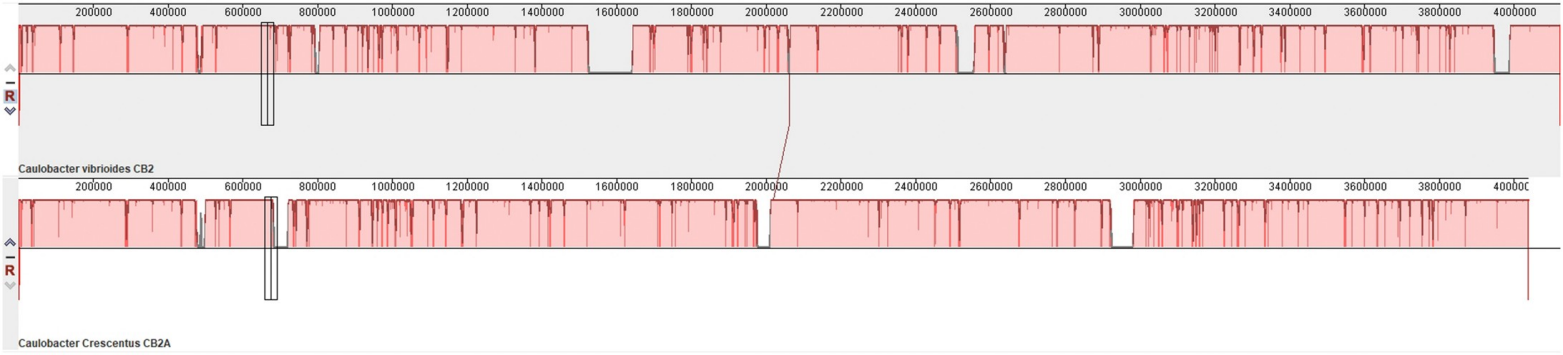
A comparison of the entire CB2 (top) and CB2A (bottom) genomes showing that small segments of the NA1000 genome are present throughout the entire CB2A genome. The horizontal red bars show that most of the CB2A genome is identical to the CB2 genome. The exceptions are the narrow regions darker red vertical lines where the CB2A genome nucleotide sequence is identical to that of the NA1000 genome. The white spaces within the lower red band that represents the CB2A genome indicate areas where the CB2A genome contains NA1000 sequences that have no corresponding region in the CB2 genome. Similarly, the gray spaces within the top red band that represents the CB2 genome correspond to regions of the CB2 genome that have been deleted from the CB2A genome.

When the CB2A genome sequence was examined, most of the genome sequence was identical to that of the CB2 genome (Fig 1). However, a careful comparison of these genomes revealed that 114 segments scattered throughout the CB2A genome were different from the corresponding segments of the CB2 genome, but they were identical to the corresponding segments of the NA1000 genome (S1 Table). Clearly, the CB2A genome is a hybrid genome, and we can only speculate about what might have happened to generate it. Some type of conjugation would be a possibility. However, the existence of a natural conjugation system was posited in 1975 [10] and never verified. I did show that conjugation could occur in the presence of plasmid RP4 [11], and we used RP4-mediated conjugation to construct a genetic map of the *C*. *crescentus* genome [12]. Our experience with conjugation is that a maximum of about 15% of the genome was transferred at one time [12]. Since CB2A contains fragments of NA1000 genome throughout its entire genome and transduction also involves small fragments of the chromosome [13], it seems more likely that some other mechanism was responsible for the uptake the observed 114 small fragments of the NA1000 genome. Regardless, both parental strains were cultured in the laboratory that isolated the CB2A strain [7], and it seems that when CB2 inadvertently came in contact with NA1000 DNA, it incorporated numerous segments of the NA1000 genome into its own genome. Interestingly, 103 of the 114 HGT insertions were small segments of DNA that included three or fewer genes. Since the CB2 genome appears to have a restriction/modification system (with an unknown sequence specificity) that is not present in NA1000 [8, 14], it is likely that most large sections of the NA1000 genome would have been cleaved by the restriction enzyme once they entered the CB2 cytoplasm. Thus, it is not surprising that most of the segments that recombined into the CB2 genome were in the range of a few hundred to a few thousand base pairs. In fact, it is possible that fragmentation of the incoming DNA might facilitate the recombination process.

**Fig 2:**
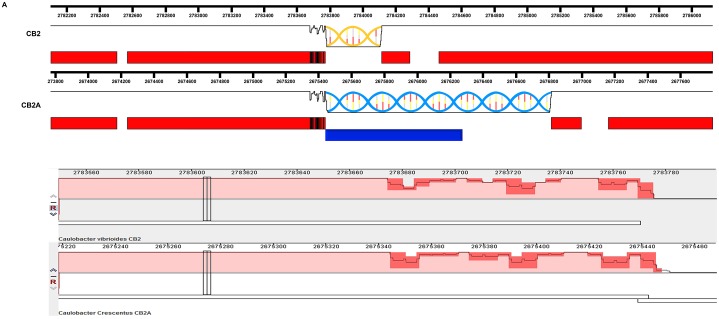
A comparison of the gene insertions in corresponding regions of the CB2 and CB2A genomes. A) The red bars correspond to genes with identical nucleotide sequences in this region of the CB2 and CB2A genomes with the vertical black bars indicating a part of the gene where the CB2A gene was identical to the NA1000 gene sequence instead of the CB2 gene sequence. The helical regions represent sequences that are unique to each genome. The blue helical region is identical to the corresponding region in the NA1000 genome, and the blue bar corresponds to a NA1000 gene that has been recombined into the CB2A genome. Thus, this NA1000 insert into the CB2A genome corresponds to the region from the leftmost vertical black bar to the right end of the blue helix. The white spaces between adjacent horizontal red bars in each genome represent intergenic regions that have identical nucleotide sequence in both the CB2 and CB2A genomes. The red regions separating the vertical black bars represent small regions where all three genomes are identical. B) A magnification of the region containing the vertical black bars in part A. The darker red regions containing the black line correspond to nucleotide sequence differences between the CB2 and CB2A genomes. The depth of the black line corresponds to the average percent identity of a sliding window comparing the two genomes. The CB2A nucleotide sequence in this region and the region to the right of it (the helix in part A) is identical to the corresponding region of the NA1000 genome. The sequences of the CB2 and CB2A genomes are identical in the region to the left of the darker red region.

Since we know the nucleotide sequences of the two parent genomes that produced the CB2A hybrid genome, a comparison of the three genomes presented a unique opportunity to study the processes that resulted in more than 100 separate recombination events. Because the two parent genomes differ at 2% of the nucleotides in their shared regions of the genome [8], the 114 regions that were identical to the corresponding segments of the NA1000 genome were well defined and easily identified as illustrated in Fig 2. When the borders of each of the 114 NA1000 regions that had recombined into the CB2A genome were examined, no new mutations were present. Every base pair on one side of the border region matched those in one of the two genomes and then abruptly switched and began matching every base pair in the other genome when the border was crossed. Thus, no new mutations were observed even though 114 double recombination events had occurred. This absence of new mutations presents strong evidence that recombination in *Caulobacter* rarely, if ever, results in new mutations. If we assume that the mechanisms of recombination that produced the hybrid CB2A genome are not different than those that are involved in normal recombination events, then the proposed [15, 16] GC-biased repair of heteroduplexes should have resulted in a small number of new AT->GC mutational events during the recombination processes that produced the hybrid CB2A genome. Since no mutations of any type were observed, it appears that mutations rarely, if ever, occur as part of the recombination process. Therefore, the GC-biased gene conversion that is well documented in eukaryotes [17, 18] does not seem to be involved in maintaining the high GC content of *Caulobacter* genomes.

The locations of the NA1000 insertions within the CB2A genome were also quite surprising. Nearly all (106 of 114) of the NA1000 insertions occurred in regions where the two parent genomes contained unique genes relative to each other. I observed that these 106 NA1000 insertions introduced 279 new genes into the CB2A genome, while replacing 328 CB2 genes (S1 Table). Thus, recombination probably occurred in the homologous regions that were hundreds of base pairs in length and flanked the non-homologous regions in the two genomes as illustrated in Fig 2. A similar observation was made when the parental CB2 and NA1000 genomes were compared to each other. About 8% of the NA1000 genes are not present in the CB2 genome, and the CB2 genome contains a similar number of genes that are not present in the NA1000 genome [8]. These unique genes are located in 260 non-homologous regions. Closer inspection revealed that only 109 of these non-homologous regions could have been due to a simple insertion or deletion event that occurred on one of the two chromosomes. The remaining 151 non-homologous regions were the result of a more complex event where a new segment of DNA appears to have replaced the original segment of DNA during the HGT event that must have occurred at each of these 151 sites. Together these data indicate that when HGT was observed either in CB2A or in the CB2/NA1000 comparison, the incoming DNA segment replaced a resident DNA segment at the majority of the HGT sites. This gain and loss of DNA segments could have occurred sequentially in the CB2/NA1000 comparison since approximately 25 million generations have occurred since they shared a common ancestor [8]. However, the recombination of NA1000 DNA into the CB2 genome produced the CB2A genome very quickly. Therefore, I propose that during HGT, the incoming segment replaces a non-homologous segment of host DNA most of the time in *Caulobacter* genomes and perhaps, in the genomes of most other bacteria.

In contrast, gene replacement did not occur in seven of the 11 instances when a tRNA gene was present at the site of recombination when the NA1000 and CB2 genomes were compared, or in any of the seven instances when an HGT event occurred at the site of a tRNA gene in the CB2A genome (S1 Table). In 16 of these 18 instances, an integrase or a transposase gene also was present, suggesting that HGT events that occur at the site of a tRNA gene may occur via a different mechanism compared to the majority of HGT events. In fact, Castillo et al. [19] described a mobile element in *Acidithiobacillus* that integrates at the site of a tRNA gene. Thus, tRNA genes may be sites that are specifically targeted by some integrases and recombinases.

Our data suggest that HGT is most commonly an exchange of DNA segments where the incoming DNA segment replaces a segment of the host genome. This new conception of HGT is consistent with several recent observations. For example, Sela et al. [3] modeled microbial genome evolution and concluded that on average, the acquisition of a gene was beneficial, but that it must be balanced by a bias towards gene deletion. Similarly, Bolotin and Hershberg [5] concluded that gene loss was not due to changes in selection since it occurred in a more continuous, clock-like manner. The data presented here indicate that a separate mechanism for gene deletion is not needed since gene acquisition and loss appear to be part of the same process. Based on these data, I propose a model for HGT-mediated recombination that is similar to the standard procedure for knocking out a gene by transforming in a selectable marker in place of a portion of the gene. Initially, regions of homologous double-stranded DNA would align, and then the CB2A data indicate that the recombination process would be initiated preferentially in regions where non-homologous regions containing up to three genes are flanked and anchored by two aligned homologous regions. Once a recombination complex is formed, the incoming DNA segment then replaces the resident segment via a double crossover. The unique aspect of this HGT model is that DNA fragments containing non-homologous regions that are flanked by regions of homology would be preferentially incorporated into the recipient genome.

**Fig 3:**
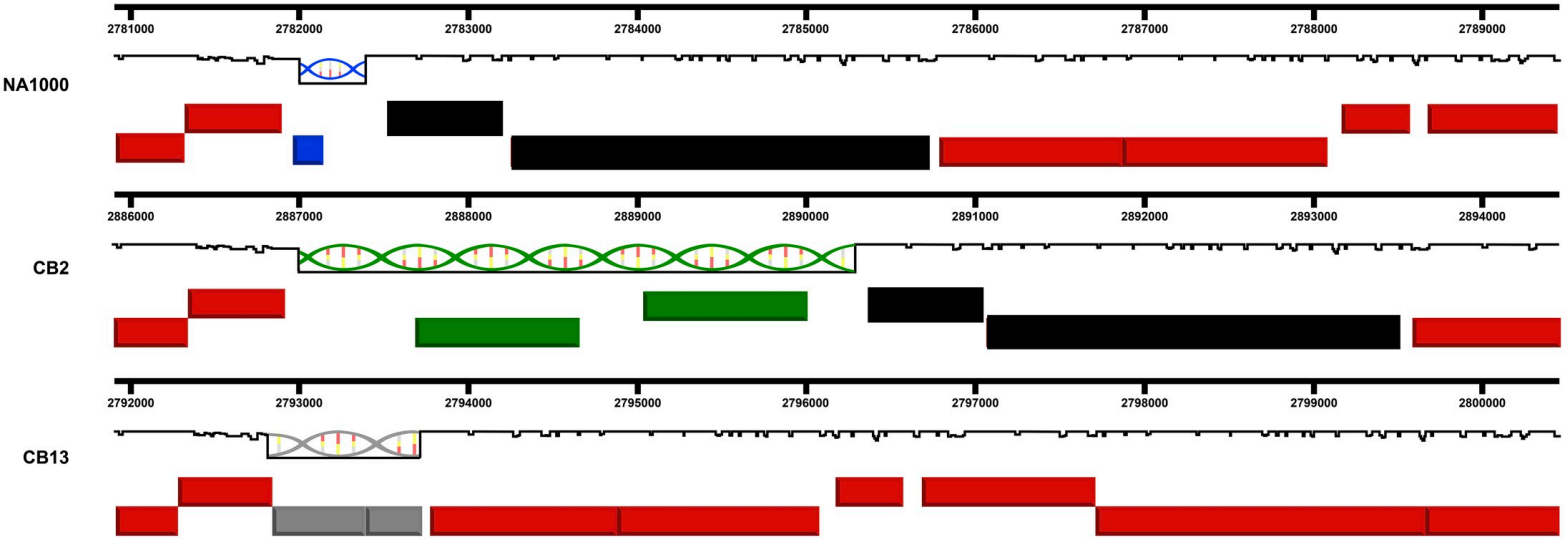
A comparison of the corresponding regions of the CB2, NA1000, and CB13 genomes. The red rectangles represent genes in shared regions of the three chromosomes. The helices represent three different insertions at the same location in each genome. The genes represented by the black rectangles are not present on the CB13 genome. The blue, green, and gray rectangles represent genes that are present in only one of the three genomes.

To test this hypothesis further, I compared the more distantly related *C*. *crescentus* CB13 genome [8, 20] to the CB2 and NA1000 genomes. I was able to identify 89 small non-homologous regions (containing fewer than 4 genes) where the corresponding DNA segment was different in all three genomes as illustrated in Fig 3. This result confirms the idea that these regions are preferential locations for HGT since at least two separate HGT events must have occurred at each of these 89 locations in order for each of the three genomes to contain segments of DNA that were not present in the other two genomes.

To determine, if this model of gene replacement by HGT applies to other types of bacteria, I compared the non-homologous regions in three closely related genomes from each of the eight bacterial genera listed in S2 Table to determine whether unique genome sequences were present at each of the HGT replacement sites. In the comparisons of each set of genomes, 23% to 56% of all of the insertion sites contained different DNA segments in either two or all three of the genomes that I examined. A similar result was obtained when we compared two Archaea genomes as well (S2 Table). HGT regions where each genome contains extra sequences either could be due a simultaneous insertion and deletion event or to two or three independent insertions at the same location. In all cases, the initial steps would involve a region that includes non-homologous DNA in the initial alignment of the homologous region of the incoming DNA to the recipient chromosome. Thus, the phenomenon of HGT involving both a region of homology and a non-homologous region occurs in a wide variety of prokaryotes.

Why would prokaryotic HGT involve simultaneous gene acquisition and gene loss? Obviously gene acquisition is beneficial because it provides a way for a bacterium to obtain genes that might allow it to compete with its neighbors more efficiently. Simultaneous gene loss also would be beneficial since it would prevent genomes from growing too large and more costly to maintain. In addition, since non-homologous regions appear to promote recombination, recently acquired genes would be deleted preferentially, an advantage since most recently acquired genes provide little if any benefit to the host cell [6]. Perhaps more importantly, if HGT occurs primarily in regions where each genome contains unique genes as it clearly did in the CB2A genome, then it would be less likely to occur in regions containing the essential genes that are present in every genome. Thus, the essential genes would be protected from loss or disruption by incoming DNA.

In addition to observing the simultaneous gain and loss of genes, I did not observe significant differences in GC content in most regions where HGT events had occurred. The exceptions were the regions mentioned above that included phage genes or mobile elements (S1 Table). These regions often had a much lower GC content. In contrast, most other insertions contained DNA segments that were similar to those present in the same genus or in closely-related genera that have a similar genomic GC content. Thus, HGT does not seem to have a significant impact on genomic GC content. This conclusion is consistent with the observation from other studies that the DNA segments present in HGT insertions are acquired primarily from members of the same genus or from closely related genera [4–6]. Also, consistent with the HGT recombination model, incoming DNA from close relatives would contain the flanking homologous regions that are needed for recombination to occur.

## Conclusion

HGT in many prokaryotes is most often an exchange of genetic material where the newly acquired DNA replaces previously acquired DNA rather than the simple acquisition of new DNA segments. This model for HGT explains how prokaryotes can keep their genomes from becoming too large while adding new genes, and it also indicates that HGT occurs preferentially at a limited number of sites in prokaryotic genomes.

## Supporting information

S1 Table Insertions from NA1000 into the CB2A genome. (XLSX)

S2 Table Evidence of site specific HGT in other prokaryotes. (DOCX)
