# Supplementary material for: Recombination and gene loss occur simultaneously during bacterial horizontal gene transfer
Source: PLoS One. 2020 Jan 28;15(1):e0227987. doi: 10.1371/journal.pone.0227987 (PMC6986707; doi:10.1371/journal.pone.0227987)
Supplement: S2 Table — (DOCX) [file pone.0227987.s002.docx]

**Supplementary Table 2. Evidence of site specific HGT in other prokaryotes.**

| **Bacteria*** | **Number of insertion sites** | **% Sites with Multiple Insertions** |
| --- | --- | --- |
| *Bacillus subtilis* | 98 | 23 |
| *Bacillus cereus* | 193 | 26 |
| *Pseudomonas putida* | 224 | 34 |
| *Escherichia coli* | 281 | 26 |
| *Synnechococcus sp.* | 486 | 38 |
| *Prochlorococcus marinus* | 50 | 56 |
| *Haemophilus influenza* | 110 | 24 |
| *Flavobacterium psychrophilum* | 24 | 25 |
| **Archaea*** |  |  |
| *Methanococcus maripaludis* | 86 | 22 |
| *Thermococcus sp.* | 253 | 62 |

*Accession numbers: *P. putida* F1 CP000712, ND6 CP003588, DOT-T1E CP003734; *Synechococcus sp.* CC9902 CP000097.1, BL107 NZ_DS022298.1, CC9605 NC_007516; *E. coli* K-12 NC_000913, 1428 CP023366, O104 CP003289; *B. subtilis* 168 NC_000964, BSP1 CP003695, RO-NN-1 CP002906; H. influenza KW20 NC_000907, 2019 CP008740, 3031 NC_014920; *B. cereus* 14579 NC_004722, FORC 013 CP011145, HN001 CP011155; *F. psychrophilum* FPG101 CP007206, JIP02/86 NC_009613, CSF259-43 NZ_CP007627, V4-24 CP008881; *P. marinus* MIT9202 DS999537, MIT9215 CP000825, MIT604 CP007753; *M.* *maripaludis* S2 BX950229, X1 CP002913, C7 CP000745; *Thermococcus sp.* 5-4 CP021848, 4457 CP002920, AM4 CP002952
